# Emotional Distraction in the Context of Memory-Based Orienting of Attention

**DOI:** 10.1037/emo0000506

**Published:** 2019-01-24

**Authors:** Sophie-Marie Raeder, Jessica K. Bone, Eva Zita Patai, Emily A. Holmes, Anna Christina Nobre, Susannah E. Murphy

**Affiliations:** 1 Oxford Centre for Human Brain Activity, Department of Psychiatry, Wellcome Centre for Integrative Neuroimaging, Oxford Neuroscience, and Department of Psychiatry, University of Oxford; 2 Oxford Centre for Human Brain Activity, Department of Psychiatry, Wellcome Centre for Integrative Neuroimaging, Oxford Neuroscience, and Brain and Cognition Laboratory, Department of Experimental Psychology, University of Oxford; 3 Oxford Centre for Human Brain Activity, Department of Psychiatry, Wellcome Centre for Integrative Neuroimaging, Oxford Neuroscience, and Brain and Cognition Laboratory, Department of Experimental Psychology, University of Oxford, and Institute of Behavioural Neuroscience, University College London; 4 Division of Psychology, Department of Clinical Neuroscience, Karolinska Institutet; 5 Oxford Centre for Human Brain Activity, Department of Psychiatry, Wellcome Centre for Integrative Neuroimaging, Oxford Neuroscience, Department of Psychiatry, and Brain and Cognition Laboratory, Department of Experimental Psychology, University of Oxford; 6 Oxford Centre for Human Brain Activity, Department of Psychiatry, Wellcome Centre for Integrative Neuroimaging, Oxford Neuroscience, and Department of Psychiatry, University of Oxford

**Keywords:** anxiety, memory-guided orienting, threat bias, attention, long-term memory

## Abstract

Attention can be guided by expectations stemming from long-term memories. In addition to such endogenous cues, exogenous salient stimuli capture attention, such as those conveying threat. This study examined the extent to which threatening distractors affect the employment of memories in guiding attention, and whether this is affected by trait anxiety. Emotional distractors were incorporated into a speeded target detection task, in which memory cues were presented simultaneously with task irrelevant emotional faces. Fearful face distractors disrupted target detection significantly more than neutral faces and the additional disruption to task performance from fearful compared with neutral faces was positively correlated with trait anxiety scores. The current findings of attentional capture by threat in the context of a second, powerful endogenous driver of attention underscore the magnitude of anxiety-related attention to threat. That is, threatening stimuli are sufficiently salient to induce prolonged disruption to goal directed behavior in anxious individuals.

Selective attention to threatening information facilitates the rapid detection of potentially threatening stimuli and the execution of appropriate responses (Mogg & Bradley, 1998; Öhman, Flykt, & Esteves, 2001). While prioritizing attention to potentially threatening environmental stimuli has clear evolutionary value, cognitive and clinical psychology models propose that excessive attentional biases toward threat may contribute to the etiology and maintenance of clinical states of anxiety (Mathews & Mackintosh, 1998; Mogg & Bradley, 1998; Williams, Watts, MacLeod, & Mathews, 1988). Excessive vigilance to threat can distort perception and can play both a causal and reinforcing role in anxiety disorders (Matthews & MacLeod, 1994, 2002).

It is possible to experimentally model selective attention to threat, which allows for the careful characterization of anxiety-related abnormalities in threat processing (Cisler & Koster, 2010; Yiend, 2010). A commonly used paradigm is the attentional-probe task (MacLeod, Mathews, & Tata, 1986; Mogg & Bradley, 1998), in which a probe replaces one of a pair of briefly presented threatening and neutral stimuli. An attentional bias toward threat is indexed by reduced RTs to probes replacing threatening versus nonthreatening stimuli. Such paradigms have demonstrated that anxious individuals detect threatening stimuli more readily and show subsequent impairment in disengaging from threat (Cisler & Koster, 2010; MacLeod & Mathews, 2012).

Studies in which threat biases are directly modified through training procedures have established the critical role played by preferential attention to threat in the cause and maintenance of anxiety disorders (MacLeod, Rutherford, Campbell, Ebsworthy, & Holker, 2002). Such work has also been instrumental in identifying specific cognitive targets of therapeutic interventions and in developing novel treatment approaches, such as attentional bias modification (Bar-Haim, 2010; Clarke, Notebaert, & MacLeod, 2014; Hakamata et al., 2010; MacLeod & Holmes, 2012; MacLeod & Mathews, 2012).

While cognitive paradigms such as the attentional-probe task have played an important role in our understanding of the threat biases associated with anxiety, they have been criticized for a lack of sensitivity (Sigurjónsdóttir, Sigurðardóttir, Björnsson, & Kristjánsson, 2015) and reliability (Grafton et al., 2017; Schmukle, 2005; Staugaard, 2009). Furthermore, the reliance on a limited set of methods to assess biased attention to threat may have hampered our understanding of the cognitive processes that are disrupted in anxiety. There remains a need for the development of additional and diverse methods to assess dysfunctional attention to threat in order to fine-tune theories, reveal mechanisms, and develop more effective cognitive treatments.

Existing paradigms typically examine the influence of threat on attention in isolation. For example, the attentional-probe task presents competing stimuli of varying valence to assess their relative pull on attention. However, outside controlled laboratory settings, attention is not exclusively guided by threat but is driven by a myriad of competing biases. Our cognition is governed by current goals, and attention is consequently guided to optimize the fulfillment of these goals. Expectations about relevant upcoming events facilitate the perceptual analysis and selection of relevant stimuli by activating goal-related schemata in working memory and setting in motion anticipatory and preparatory functions (Desimone & Duncan, 1995; Gazzaley & Nobre, 2012; Theeuwes, Belopolsky, & Olivers, 2009). Furthermore, long-term memories provide an additional source of predictions that significantly enhance behavioral performance and neural processing of target stimuli occurring within learned, anticipated contexts (Chun & Jiang, 1999; Summerfield, Gitelman, Mesulam, & Nobre, 2006).

Exogenous threat distraction is likely to operate in the context of nonemotional endogenous cognitive biases that are deployed to support goal attainment. As threat has an exaggerated pull on attention in anxious individuals, it is plausible that the perceptual salience of threatening stimuli interferes with expectation-driven attention selection and that the magnitude of such interference correlates positively with trait anxiety. There are existing paradigms that examine threat biases in the context of emotion-related interference of the employment of expectations. For example, the Posner-style emotional spatial cueing paradigm presents neutral and threatening spatial cues that either validly or invalidly predict an upcoming target location (Fox, Russo, Bowles, & Dutton, 2001; Posner, 1980). Previous studies using this paradigm have demonstrated threat biases in anxious individuals, indicated by quicker responding to valid threat trials coupled with slowed responding to invalid threat trials, compared to benign-cued trials (Bar-Haim, Lamy, Pergamin, Bakermans-Kranenburg, & van IJzendoorn, 2007; Cisler, Bacon, & Williams, 2009; Mogg, Holmes, Garner, & Bradley, 2008). Such tasks exploit external cues, which are internalized and used to predict task-relevant events. However, most endogenous drivers of visual attention are intrinsic to a context, based on expectations built upon prior experience, stored within memories. For example, it has been established that long-term memories for target locations within previously encountered scenes can enhance perceptual sensitivity and response speeds to identify and discriminate targets in those scenes (Patai, Doallo, & Nobre, 2012; Summerfield et al., 2006). Considering the effect of threat on the processing of task-relevant endogenous signals in the Posner-style task, an appropriate follow-up enquiry is whether threatening stimuli may similarly affect the employment of learned intrinsic nonemotional cognitive biases; and whether this is exacerbated in, or exclusive to, anxious individuals.

The current study therefore investigated the effect of emotionally salient distractors on memory-guided attentional orienting, as well as the dependence of these effects on individual differences in trait anxiety. Our study complements investigations that have considered how emotional content intrinsic to material to be learned affects subsequent memory (e.g., Buratto, Pottage, Brown, Morrison, & Schaefer, 2014; Srinivasan & Gupta, 2010). Our interest was in understanding whether distraction by emotional material interacted with everyday nonemotional, memory-based attentional biases, which we propose to be pervasive and highly adaptive in guiding performance (Chun & Turk-Browne, 2007). In order to investigate this, emotional distractors were incorporated into an existing memory-guided orienting task (Patai et al., 2012). In this task, participants explore visual scenes overtly to learn the unique locations of hidden predefined targets. Twenty-four hours later, participants perform a speeded target-detection task in which the previously learned scenes act as cues to orient attention based on participants’ long-term memories of the target location in each scene. Using this paradigm, previous studies have reported significant benefits in the perceptual sensitivity and speed to identify and discriminate targets on valid, compared to invalid trials (Salvato, Patai, & Nobre, 2016; Summerfield et al., 2006; Summerfield, Rao, Garside, & Nobre, 2011), demonstrating that participants’ memories were effectively used to guide attention to learned target locations.

The current study adapted this paradigm to include emotional distractors, which were presented simultaneously with the memory cues. A single fearful or neutral face was embedded in the cue scenes, and the extent to which the fearful distractors influenced subsequent target detection, as compared to neutral distractors, indexed emotion-related distraction. Thus, we were able to investigate the extent to which threat distractors captured attention in complex scenes so as to disrupt memory-based attention. We predicted that fearful faces would capture attention to a greater extent and consequently be more disruptive to task performance than neutral faces. Moreover, given that contemporary cognitive models of anxiety suggest that it is characterized by an overdominance of exogenous, bottom-up drivers of attention, and impaired regulation of this by endogenous, top-down, goal-directed attentional control mechanisms (Eysenck, Derakshan, Santos, & Calvo, 2007), we predicted that the degree of distractor disruption was expected to be positively associated with trait anxiety.

## Method

### Participants

Thirty-two participants (22 females, 10 males), aged 19–26 years (mean: 20.9 years), were recruited through an online participant database and through online adverts and local flyers. The current sample size is comparable to other studies investigating anxiety-related threat biases, particularly those similarly using median-split analyses (Fox, 2008; Koster, Crombez, Verschuere, Van Damme, & Wiersema, 2006). All participants had normal or corrected vision, had no history of neurological or psychiatric illness, and were fluent in English. Participants gave their written informed consent and were compensated £10/hr for their participation. The study was approved by the Oxford University Central University Research Ethics Committee (MSD-IDREC-C1-2014–075).

### Procedure

Participants attended two test sessions at the Department of Psychiatry at the University of Oxford, conducted 24 hr apart. In the first session, participants completed the trait anxiety scale of the Spielberger State–Trait Anxiety Inventory (STAI-T; Spielberger et al., 1983), the Beck Depression Inventory (BDI-II; Beck, Brown, & Steer, 1996) and the Neuroticism subscale of the Eysenck Personality Questionnaire (EPQ-N; Eysenck & Eysenck, 1975). They then completed the Learning Task (see below). The following day, participants returned to complete the Orienting and Memory Tasks, which were always completed in this order.

### Stimuli

#### Scenes and keys

**Figure 1 fig1:**
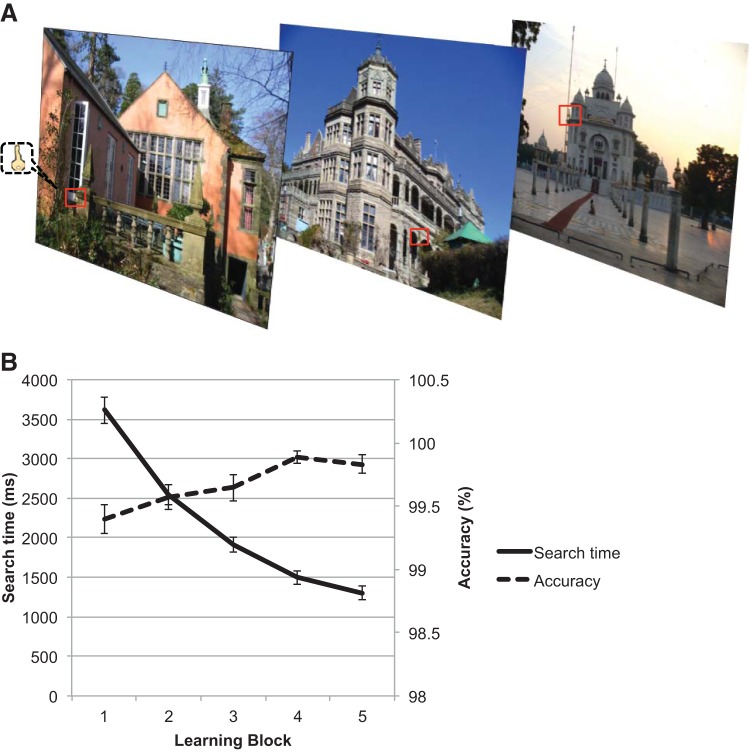
Learning Task: design and results. (A) Participants overtly searched for keys, located in one of four quadrants, for 168 naturalistic scenes (three examples shown, red box for illustrative purposes only. (B) Over the course of learning blocks, participants became more accurate and quicker at detecting targets.

168 everyday scenes (see Figure 1A for examples) were taken from an existing database obtained collectively by the laboratory. The set consisted of 35 indoor scenes and 133 outdoor scenes with varied content. The presence of people was kept at a minimum, as was the use of large, salient objects in the foreground. Given their intended naturalistic quality, there was inevitable variability across scenes. However, by counterbalancing the scenes across experimental conditions, we were able to ensure that intrinsic characteristics of the scenes did not affect results. Scene stimuli will be made available on request. Scenes were sized 1000 × 750 pixels and subtended 22° × 17° of visual angle when viewed from 100 cm. In the Learning Task (see Figure 1A), a gold key (15 × 29 pixels, equivalent to 0.3° × 0.7°) was embedded in one of the four quadrants of the scene. In the Orienting Task, the key was larger and brighter (25 × 49 pixels, equivalent to 0.6° × 1.1°) to enhance contrast and thus prevent chance-level accuracy rates.[Fig-anchor fig1]

#### Faces

**Figure 2 fig2:**
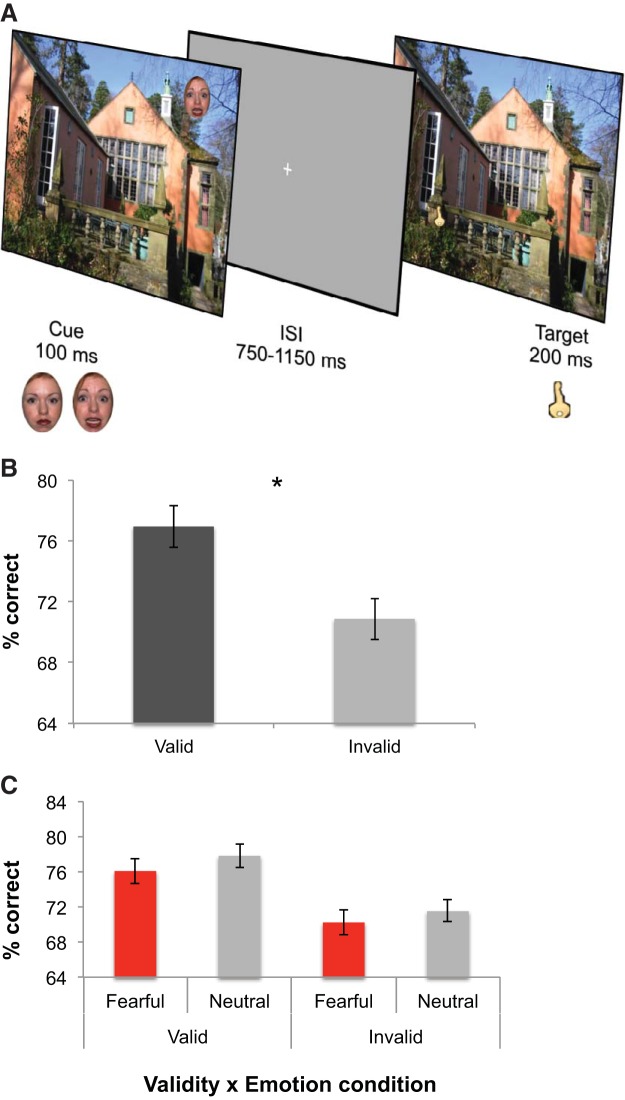

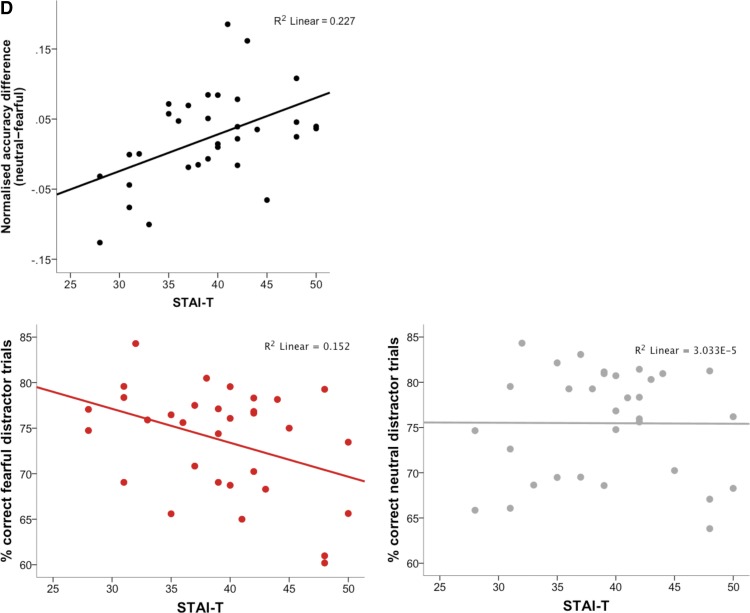
Orienting Task: design and results. (A) Cue scenes containing either fearful or neutral faces were presented for 100 ms. After an ISI ranging from 750 to 1150 ms, target scenes, which presented the key on half the trials, appeared for 200 ms. (B) All participants showed a significant validity effect, whereby they exhibited greater accuracy for valid versus invalid trials. Error bars represent standard errors of the mean. Asterisks indicate significant differences between valid and invalid conditions (*p* ≤ .05). (C) While valid trials yielded overall greater accuracy, results showed no Validity × Emotion interaction. (D) There was a significant positive correlation between STAI-T scores (X axis) and mean accuracy difference scores (top). Separate correlations for each emotion condition confirmed that STAI-T was significantly negatively correlated with accuracy on trials containing fearful distractors (bottom left), while STAI-T did not correlate with accuracy on trials with neutral distractors (bottom right).

A total of 168 colored face stimuli were used in the Orienting Task. The face stimuli were taken from the Amsterdam Dynamic Facial Expressions Set (17 faces; van der Schalk, Hawk, Fischer, & Doosje, 2011), Pictures of Facial Affect (13 faces; Ekman & Friesen, 1976), the Radboud Faces Database (15 faces; Langner et al., 2010) and the NimStim Set of Facial Expressions (39 faces; Tottenham et al., 2009). Eighty-four different face identities were used (50 male), each with a fearful and a neutral expression (see Figure 2A for examples). A standardized oval was used to crop the faces to ensure that the faces were of a standard size across trials. The oval was placed so as to include only the eyes, nose and mouth, part of the chin, and the top of the hairline. Faces were 91 × 140 pixels (equivalent to 2.1°x 3.2° when viewed from 100 cm). An additional face, taken from the Facial Expressions and Emotion Database of the Technical University Munich (Wallhoff, 2005), was used for the practice trials of the Orienting Task. This face was pixelated using Adobe Photoshop (version CS6), creating a 91 × 140-pixel oval.[Fig-anchor fig2]

All data and materials have been made publicly available via the Open Science Framework and can be accessed at https://osf.io/gz3vb/.

### Tasks

The entire memory-guided orienting experiment consisted of three experimental phases: a Learning Task; an Orienting Task; and a Memory Task.

#### Learning Task

The Learning Task was based on that reported by Patai and colleagues (2012). Participants were instructed to search for a small gold-colored key (target) in each of 168 scenes. Scenes were viewed one at a time, and participants searched for the key embedded in one of the four quadrants (Figure 1A). They were free to move their eyes (overt search). Once they had located the key, participants responded by clicking the mouse to activate the cursor and then positioning it on the key location. Following the response, visual written feedback (1000 ms) was provided (i.e., *key found* or *key not found*) on each trial. Scenes remained on the screen for 2 min or until a response was made. If participants did not respond within 2 min, they received feedback reading *key not found*, followed by the start of the next trial.

After searching for the key in all 168 scenes, participants took a brief break and then repeated the procedure for the same set of scenes. In total, participants completed the procedure five times. The key remained in the same location within each scene for each of these five learning blocks, thereby allowing participants to form spatial-contextual memories of the location of the key in each scene. The presentation order of scenes was randomized in each block. Written feedback was presented at the conclusion of each block, indicating the total fraction of keys found. Search times and accuracy were recorded. A circle with a radius of 50 pixels around the target (key) defined the region within which responses were deemed accurate. Scenes for which participants did not locate the key in two or more blocks were excluded from further analyses.

#### Orienting Task

The Orienting Task was performed 24 hr after the Learning Task (see Figure 2). Participants were instructed to maintain central fixation during each trial by continually fixating on a central cross, which remained on the screen throughout the entire task. Participants were asked to try to refrain from blinking during scene presentations.

Participants first completed a practice session containing 12 novel scenes with a distracting scrambled face. Using a scrambled face during the practice session allowed participants to become accustomed to the appearance of distractor stimuli without habituating to face valence. Participants were first shown a novel cue scene for 100 ms, featuring the scrambled face. After an interstimulus interval (ISI) ranging between 750 and 1150 ms, the same scene reappeared for 200 ms; the key was present in half of these target scenes in a randomly varied location. Participants had to indicate whether the key was present or absent by making a forced-choice response using the mouse. After completing the practice session, participants were given the opportunity to clarify any outstanding questions and then started the task.

In the experimental task, each trial began with the brief presentation of a cue scene, which was a scene that participants had previously been exposed to during the Learning Task. This cue scene was presented for 100 ms and had a fearful or neutral face embedded in it. The key was never present in the cue scene. After a random ISI ranging from 750 to 1150 ms, the same scene was presented again without the face for 200 ms. On half of the trials (i.e., on 84 trials), this scene contained the target key, which was located in either a valid (learned) or in an invalid (novel) location in the opposite hemifield. Trials in which the key was present were equally divided into valid and invalid trials (42 trials each). On the remaining 84 trials, the key was not present in the scene. Participants were asked to indicate whether the key was present or absent via a left or right mouse click, respectively, and were given a 1000 ms time window to respond. After a random intertrial interval of 750 to 1150 ms, the next trial began. Self-timed breaks were provided after every 14 trials. During the cue scene, faces were always located in a different quadrant from both the learned (valid) and novel (invalid) key location. That is, faces never cued valid or invalid key locations and were therefore always distractors (Figure 2A). Participants were told that the faces were not relevant to the task and were instructed to ignore them.

All 168 scenes from the Learning Task were included in the Orienting Task and the assignment of each scene according to the experimental conditions of target presence (present, absent), validity (valid, invalid), and face emotion (fearful, neutral) was counterbalanced across participants, resulting in eight versions of the task. Accuracy of target detection and reaction time (RT) were used as outcome measures on this task. Individual raw RTs above or below three standard deviations across conditions were excluded from the analyses for each participant. This resulted in the removal of 2.6% of trials. Trials removed from RT analyses on this basis were also excluded from the accuracy analyses.

#### Memory Task

**Figure 3 fig3:**
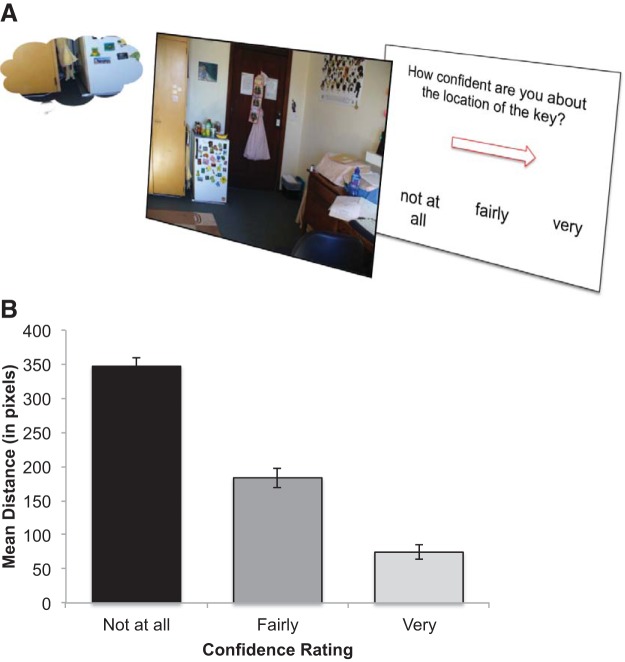
Memory Task: design and results (A) Participants indicated the learned location of the key, followed by a confidence rating. (B) Mean distance (Y axis) from target location as a function of confidence ratings (X axis). Mean distance decreased as confidence ratings increased. Error bars represent standard errors of the mean.

Following the Orienting Task, participants completed a memory recall test. In this task, participants viewed each of the 168 scenes in turn, and indicated the learned location of the key (Figure 3A). After indicating the remembered location by positioning the cursor on the location and using a mouse click to place their response, participants were asked to complete a confidence rating of their memory *(not at all, fairly,* or *very confident*), using the left, center, and right mouse buttons respectively. Feedback was not provided for memory recall. Mean distance between the original and indicated key locations was used as an index of spatial recall; this was calculated as the Euclidean distance between the X and Y coordinates of response and actual key location.[Fig-anchor fig3]

### Analyses

The data were analyzed using ANCOVA, with task conditions included as within subjects factors and trait anxiety (STAI-T) scores included as a continuous predictor.

Across analyses, where the assumption of sphericity was violated, degrees of freedom were corrected using Greenhouse-Geisser estimates of sphericity. All post hoc tests were conducted with Bonferroni correction.

## Results

**Figure 4 fig4:**
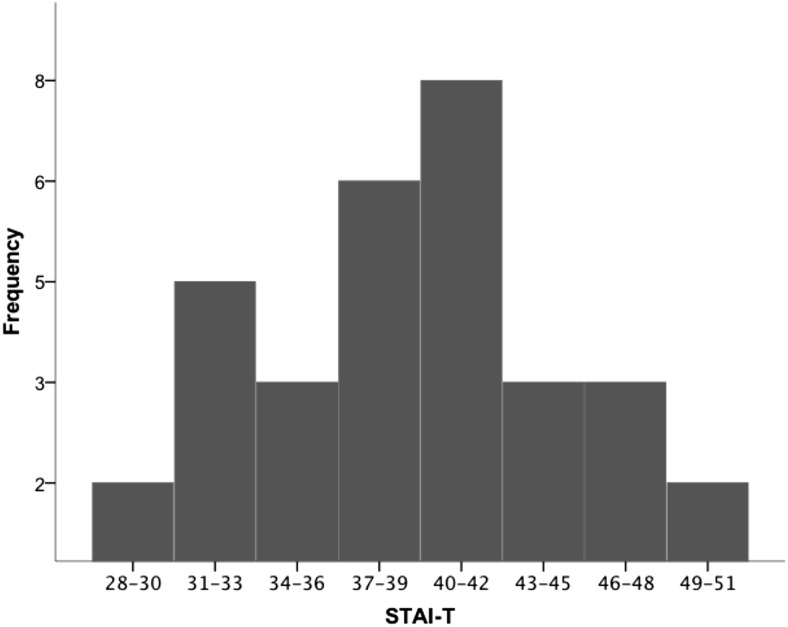
The spread of STAI-trait scores. The Shapiro-Wilk test (Shapiro & Wilk, 1965) revealed no evidence (*p* > .05) that the distribution of STAI-T scores was non-normal.

Participants’ trait anxiety scores ranged from 28 to 50 (*M* = 39.2, *SD* = 6.2, see Figure 4). These scores are similar to the published norms for this age group (*M* = 36, *SD* = 10; Spielberger et al., 1983).[Fig-anchor fig4]

### Learning Task

Search times decreased and accuracy increased with learning over the five overt-search blocks (see Figure 1B). Because of the long time allowed for searching for the target within each scene, the accuracy for locating the target key was consistently high from the first learning block (accuracy, *SD*, range: 99, 0.70, 1.80%). Nevertheless, ANOVAs testing for linear increases in accuracy showed a main effect of learning block [*F*(2.89, 83.88) = 5.62, *p* = .002, η_p_^2^ = .16] and significant linear contrast over the learning blocks [*F*(1, 30) = 17.76, *p* < .001, η_p_^2^ = .37]. Reaction times showed strong modulation over learning. There was a significant main effect of learning block [*F*(1.53, 46.03) = 159.59, *p* < .001, η_p_^2^ = 0.84] and a significant linear decrease in search times over blocks [*F*(1, 31) = 217.19, *p* < .001, η_p_^2^ = .88].

To assess potential anxiety effects, a mixed-model ANCOVA was conducted with a within-subject factor of block (1–5) and trait anxiety (STAI-T) as a continuous predictor. There was no main effect of trait anxiety or interaction between trait anxiety and block for accuracy [main effect: *F*(1, 30) = 0.49, *p* = .49, η_p_^2^ = .016; interaction: *F*(4, 120) = 0.56, *p* = .69, η_p_^2^ = .018] or search times [main effect: *F*(1, 30) = 1.30, *p* = .26, η_p_^2^ = .041; interaction: *F*(4, 120) = 1.12, *p* = .35, η_p_^2^ = .036]. Anxiety therefore did not impact the formation of new spatial-contextual associations for nonemotional targets within scenes. The comparable learning across trait anxiety provides a clean baseline for the subsequent Orienting Task, allowing for anxiety-related emotional capture effects to clearly be isolated.

One participant did not successfully locate the key in one of the scenes in 2 of the blocks and this participant’s data for this scene was excluded from further analyses.

### Orienting Task

A mixed-model ANCOVA was conducted on percent correct accuracy scores using within-subject factors of face emotion (fearful, neutral) and validity (valid, invalid), and trait anxiety as a continuous predictor. This showed a significant main effect of validity [*F*(1, 30) = 4.03, *p* = .054, η_p_^2^ = .12], reflecting increased accuracy on valid compared to invalid trials (valid: 77.6%, CI [75.4, 79.8] invalid: 71.6%, CI [69.2, 73.9]; see Figure 2B). There was also a significant main effect of emotion [*F*(1, 30) = 6.10, *p* = .019, η_p_^2^ = .17], reflecting increased accuracy on trials preceded by a neutral face compared with a fearful face (mean percent correct: neutral 75.5%, CI [73.3, 77.7], fearful 73.7%, CI [71.7, 75.7]), and a significant interaction between face emotion and trait anxiety, [*F*(1, 30) = 8.13, *p* = .008, η_p_^2^ = .21]. To clarify this face emotion x trait anxiety interaction further, the difference in mean accuracy for fearful versus neutral conditions, normalized against the overall mean accuracy across conditions, was used to test for correlations between emotional distraction and STAI –*T* scores. This yielded a significant positive correlation, *r* = .48, *p* = .006. Separate correlations between STAI-T and accuracy on trials containing fearful distractors and accuracy on trials with neutral distractors revealed that anxiety was significantly negatively correlated with accuracy on fearful distractor trials, *r* = −.39, *p* = .027, while the correlation between anxiety and accuracy on neutral distractors trials was not significant, *r* = −.006, *p* = .98 (see Figure 2D). There was no interaction between validity and emotion, *p* = .91 (see Figure 2C).

As both neuroticism and depression are often comorbid with anxiety, separate analyses were conducted using neuroticism (EPQN) and depression (BDI) scores to assess whether the observed threat distraction was additionally driven by neuroticism and depression. Two ANCOVAs were conducted on accuracy, with the respective continuous predictors of neuroticism and depression, and validity and face emotion as within-subjects factors. The analysis using neuroticism scores revealed a significant validity effect, *F*(1, 30) = 11.30, *p* = .002, η_p_^2^ = .27, and a trending interaction between emotion and neuroticism, *F*(1, 30) = 3.53, *p* = .070, η_p_^2^ = .11. The ANCOVA using depression scores showed a significant main effect of validity, *F*(1, 30) = 18.82, *p* < .001, η_p_^2^ = .39. There were no further main effects or interactions, all *p*’s > .20.

Reaction times were not of primary interest in this task, which required a forced-choice response based on a difficult perceptual discrimination. Nevertheless, RTs were analyzed for completeness. Reaction times were compared for correct trials. Analysis of RTs revealed no significant main effects or interactions (all *p*’s > .33).

### Memory Task

The mean distance between the original and indicated key location was 207.56 pixels (*SD* = 79.47). Participants’ confidence ratings varied in accordance with the mean distance between their response and the key location; as they became less confident their accuracy decreased (Figure 3B).

To determine whether distractor emotion and target validity from the preceding Orienting Task affected subsequent recall, a mixed-model ANCOVA was conducted with within-subject factors of face emotion (fearful, neutral) and validity (valid, invalid), and trait anxiety (STAI-T) as a continuous predictor. There was no significant effect of validity, emotion or trait anxiety, and no significant interactions (all *p*’s > .43). The lack of interaction between emotion and anxiety suggests that recall for fearful versus neutral faces did not differ as a function of anxiety. Further analyses also confirmed that there were no significant differences in recall on trials in which the key was absent versus present in the Orienting Task.

To complement these findings and to provide a broader index of accuracy, accuracy was indexed by quadrant identification. A mixed-model ANCOVA was conducted with target validity and distractor emotion of the preceding Orienting Task as within-subjects factors and trait anxiety as a continuous predictor. There was no significant effect of validity, emotion or trait anxiety, and no significant interactions (all *p*’s > .31).

To assess the degree to which target validity and distractor emotion from the Orienting Task affected RTs in the Memory Task, a further mixed-model ANCOVA was conducted, comparable to that above. Results showed no significant main effects of validity, emotion or trait anxiety, and no significant interactions (all *p*’s > .34).

Finally, to determine whether the location of the previous distractor faces influenced participants’ explicit memories of the target locations, we compared the incidence of participants incorrectly indicating that the memorized target location was in the quadrant previously occupied by the distractor face versus in either of the other two incorrect quadrants. A comparison of these quantities revealed no significant differences in the quantity of errors occurring in the quadrant of the face and the average quantity of errors occurring in the remaining two quadrants, *t* (31) = 0.88, *p* = .34, *d* = 0.17, CI [0.97, 2.72]. These results suggest that the location of the distractor faces did not affect participants’ explicit memories.

## Discussion

The current study investigated the effect of emotionally salient distractors on memory-guided attentional orienting, and how this is modulated by trait anxiety. Emotional distractors were incorporated into a speeded target detection task, in which nonemotional memory cues were presented simultaneously with irrelevant distracting fearful or neutral face stimuli. Consistent with our predictions, we found that fearful face distractors disrupted performance on a subsequent target detection task significantly more than neutral face distractors. Importantly, the extent to which fearful faces were more disruptive to task performance than neutral faces was positively correlated with trait anxiety scores, and further analyses highlight that this threat-related disruption to task performance was only evident in those participants with high trait anxiety scores.

Attention is guided by a myriad of competing influences. In this study, we adapted an experimental paradigm for investigating long-term memory-guided orienting of attention to incorporate emotional distractors. In this way, we were able to measure the effect of threat distraction within the context of another, endogenous, and nonemotional driver of attention and assess the relative pull of each of these on attention, indexed by task performance. Consistent with previous studies (Patai et al., 2012), we found that there was a significant effect of target validity; targets that appeared in the expected (learned) location were more accurately detected than those that appeared in a location that was incongruous with the previous learning trials.

Such an effect is a demonstration of the expected memory-guided attentional cueing, and confirms that participants were using memory cues based on their previous learning to spatially orient their attention to the expected location of the key. In addition, there was a significant effect of the emotion of the face, whereby participants were more accurate in their detection of targets in scenes that had been preceded by a neutral face distractor, than those preceded by a fearful face distractor. Thus, as predicted, the fearful face disrupted task performance to a greater extent than neutral face distractors. Interestingly the emotional-distraction effect did not significantly interact with memory-based orienting of attention, suggesting that multiple sources of biases can operate concurrently and independently to influence task performance.

Importantly, the current study demonstrated that the extent to which fearful, compared to neutral, faces disrupted task performance correlated with trait anxiety scores. Thus, the performance of participants who scored highly on a self-report measure of trait anxiety was disproportionately disrupted by fearful versus neutral face distractors, compared with low trait anxiety participants. This is consistent with previous literature demonstrating that anxious individuals more readily detect threat and show subsequent impairment in disengaging from such threat (Cisler & Koster, 2010; MacLeod & Mathews, 2012). Meta-analyses of previous studies have similarly reported such attentional biases toward threatening stimuli (Bar-Haim et al., 2007), using paradigms such as the attentional-probe task (MacLeod et al., 1986; Mogg & Bradley, 1998). Results are also in line with The Attentional Control Theory (Eysenck et al., 2007), which posits that anxiety is marked by an impaired balance between bottom-up exogenous attention and top-down endogenous attention, with the former receiving exaggerated priority. That is, anxious individuals exhibit greater influence of the stimulus-driven system coupled with deficient top-down control, resulting in impaired goal fulfillment and task performance. The current findings demonstrate such distorted weighting, with trait anxiety correlated with greater distraction by the fearful faces at the expense of task-relevant target detection, suggesting a bias toward the bottom-up salience of threat paired with weak input of top-down control.

The current paradigm expands on previous findings by placing threat biases in the context of competing nonemotional drivers of attention, thereby creating a more realistic context within which to investigate anxiety-related biases. In addition to the ecological validity accorded by modeling multiple and competing drivers of attention, the use of complex naturalistic scenes further enhances the paradigm’s realistic quality. Studying attentional orienting in contexts closely resembling our everyday environments provides a more accurate depiction of how we filter information-rich environments to direct attention toward relevant information.

It is interesting to note that there was a relatively long delay between the presentation of the cue scene, containing the face distractor, and the target presentation (750–1150 ms). Any distraction effect of fearful compared with neutral faces would therefore have had to persist across this delay period in order to influence target detection. As the fearful face was presented for only 100 ms, the observed disruption to subsequent task performance was not contingent on the continued presence of a threatening stimulus. This suggests that anxious participants continued to mentally engage with the fearful faces over an extended period of time. Studies examining attentional biases to threat often restrict their scope of investigation to reactivity to threat at early stages of processing (Schuyler et al., 2014). However, it is increasingly recognized that more *prolonged* disruption to task performance from threat distractors may be associated with anxiety, and such disruption may have a particularly impairing effect on the daily life of anxious individuals (Forster, Nunez-Elizalde, Castle, & Bishop, 2014; Schuyler et al., 2014). While early reactivity to threatening stimuli may serve a biologically adaptive purpose across all individuals, it may be equally adaptive to subsequently employ emotion regulatory processes in order to readily recover from the adverse effects of such initial threat detection (Forster et al., 2014). As such, this represents a target for improving cognitive treatments of anxiety. Given that even simple threatening stimuli of the type used here are sufficiently salient to disrupt the usage of memories in orienting attention in anxious individuals, strategies to mitigate the downstream effects in the lives of patients are much needed. Such processes may well underlie the functional impairments associated with anxiety such as lapses in concentration and impoverished performance on daily tasks. However, given the testing of healthy volunteers based only on individual differences in trait anxiety, the implications for a clinical population are limited and must be viewed with caution.

The findings must be interpreted in light of several limitations. First, the distracting stimuli in the current study were solely neutral and threatening faces, with the latter’s depicted emotion necessarily rendering them more perceptually salient. Although fear is a particularly relevant emotional category to consider in the context of anxiety, using only one category necessarily provides only limited insights into how emotional saliency biases performance in the context of anxiety. Future research should therefore include stimuli depicting other emotions (e.g., happiness, surprise) as distractor stimuli in the paradigm in order to clarify whether the anxiety-related distraction effects are specific to threat valence.

Second, the STAI-state scale was not assessed. Previous research has demonstrated a dissociation between the attentional processes targeted by trait anxiety and those affected by state anxiety. While the former modulates executive control processes, the latter affects the alerting and orienting networks of attention (Posner & Petersen, 1990; Posner, Rueda, & Kanske, 2007). The relationship between state and trait anxiety has been delineated, such that trait anxiety is marked by a predisposition to experience heightened state anxiety when confronted with threat (Endler & Kocovski, 2001). The current study found that trait anxiety modulated the degree of threat distraction. However, we cannot ascertain the extent to which state anxiety contributed to such threat distraction. Given the outlined differences in the two forms of anxiety, future research investigating the interaction of threat biases and memory-guided orienting should consider both state and trait anxiety as variables.

Finally, face stimuli from multiple databases were used as to ensure that each scene contained a unique distractor face in the Orienting Task in order to minimize habituation effects (Breiter et al., 1996; Gauthier et al., 2000). However, as this inevitably introduced variation to the stimuli set, a mixed-design ANCOVA was conducted with face sets (1–4) as the between-subjects factor and trait anxiety as the continuous predictor for accuracy (% correct) in the Orienting Task to assess whether faces from different databases led to different results in overall accuracy. Results showed no main effect of face sets, *p* = .77, suggesting that accuracy in the Orienting Task did not differ between the face sets. Further, there was no interaction with STAI-T, *p* = .83.

In summary, this study expands the investigation of anxiety-related threat biases to incorporate other endogenous drivers of attention, thus modeling vigilance to threat within the context of complex, competing sources of influence on attention. These findings situate the well-documented anxiety-related attentional biases within a relatively more ecologically valid context and lend weight to the notion that delayed recovery from threat distractors, and the resulting prolonged disruption of goal directed behavior, is aberrant in anxiety. As such, this process may be an important therapeutic target for clinical interventions.
